# Ureterocele with embedded stone suggesting a diagnosis of bladder tumor: beware of the diagnostic trap! (case report and literature review)

**DOI:** 10.11604/pamj.2024.49.119.44949

**Published:** 2024-12-13

**Authors:** Idriss Ziani, Ahmed Ibrahimi, Yassine Nouini

**Affiliations:** 1 Urological Surgery Department “A”, Rabat University Hospital, Rabat, Morocco; 2 Mohammed V University, Rabat, Morocco

**Keywords:** Holmium laser, endoscopic resection, bladder tumor, ureterocele, case report

## Abstract

Ureterocele is a congenital urinary malformation resulting in pseudo-cystic dilation of the terminal ureter under the mucosa. We present the case of a 47-year-old patient who presents with irritative symptoms of the lower urinary tract with hematuria. Initial imaging suggested a bladder tumor with a bladder stone, before correcting the diagnosis of ureterocele with embedded stone by cystoscopy. Treatment included endoscopic ureterocelotomy with laser stone fragmentation.

## Introduction

The ureterocele is a pseudo-cystic dilatation of the terminal ureter under the mucosa. It is a rare malformative uropathy especially if it occurs in a simplex ureter. Its symptoms are sometimes misleading [1]. As for treatment, the endoscopic approach is increasingly used [1]. The aim of this article is to illustrate with a clinical case the difficulties of diagnosing this uropathy, hence the interest in bringing together the results of several explorations before the therapeutic decision [2].

## Patient and observation

**Patient information:** we report a case of a 47-year-old patient who consulted for irritating symptoms of the lower urinary tract associated with a few episodes of hematuria.

**Clinical findings:** the clinical examination was unremarkable, microscopic hematuria was observed on the urine strip.

**Timeline of the current episode:** the symptoms had been evolving for 6 months with a gradual exacerbation.

**Figure 1:**
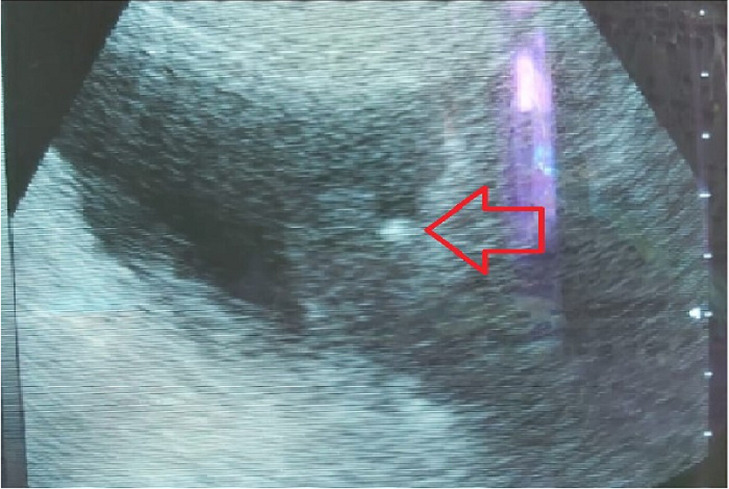
ultrasound aspect showing a bladder process with a bladder stone

**Figure 2:**
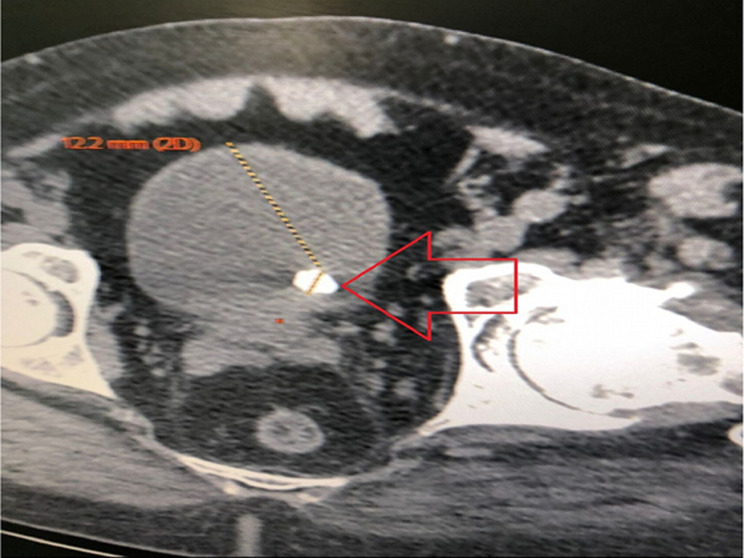
computed tomography scan showing a bladder stone with wall thickening of the bladder

**Diagnostic assessment:** ultrasound exploration revealed a left lateral bladder lesion process with an embedded stone (Figure 1), and the computed tomography (CT) scan revealed 12 mm lithiasis with suspicious tissue thickening (Figure 2).

**Diagnosis:** radiological exploration concluded that there was a suspicious bladder mass associated with bladder lithiasis; this required a comparison with the endoscopic data.

**Figure 3:**
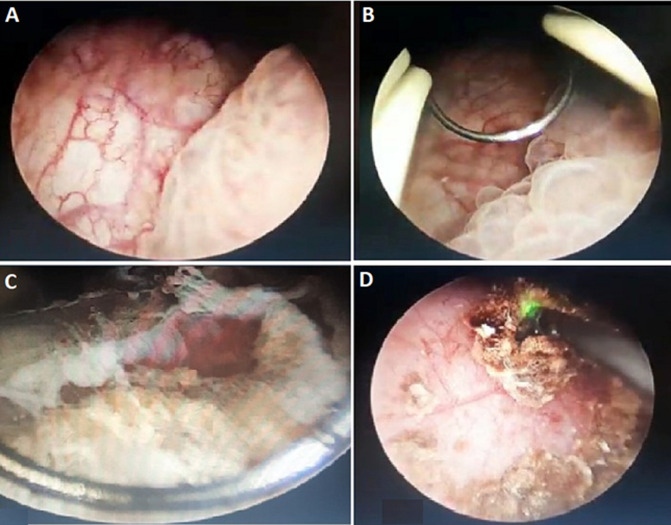
A) left lateral bladder mass; B) endoscopic resection of the mass; C) visualization of the stone in the ureterocele; D) endoscopic fragmentation of the stone by laser

**Therapeutic intervention and follow-up:** an endoscopic exploration for diagnostic and therapeutic purposes was considered and revealed a tissue lesion with an inflammatory appearance, his endoscopic resection revealed a stone embedded in a ureterocele. The therapeutic approach consisted of resection of the ureterocele and then fragmentation of the stone using the holmium laser at the same time of operation (Figure 3). The resected tissue was analyzed by pathology examination which did not show any sign of malginity.

**Prognostic characteristics:** the prognosis for ureterocele is good after treatment, there are no notable complications.

**Follow-up and outcome of interventions:** the postoperative course was simple, allowing discharge home after removal of the bladder catheter on day 1. The check-up at 3 months and 6 months took place without any abnormality.

**Patient perspective:** the patient was pleased with the small incision required for the minimally invasive treatment and the noticeable reduction in clinical symptoms.

**Informed consent:** the patient gave his consent to the exploration.

## Discussion

Ureteroceles are congenital uropathies generally diagnosed during prenatal ultrasounds or early childhood assessments and are a rare phenomenon. However, there are exceptional cases where symptoms related to ureterocele can manifest later in life, which poses a diagnostic difficulty [3]. The risk of obstructive uropathy-induced chronic kidney damage necessitates treatment. Depending on the patient's age, the clinical presentation of ureteroceles can vary significantly due to the wide range of symptoms that are associated with the condition [4]. Diagnostic imaging studies, such as uroscan and urinary tree ultrasound, are crucial; in cases of doubt, cystoscopy is still required [5].

The endoscopic method enables endoscopic management of the ureterocele as well as diagnostic confirmation [5]. Transurethral incisions are still the current standard of care for ureteroceles [5]. Although some cases continued to exhibit mild vesicoureteral reflux even after intervention, this approach was found to be safe and effective [5].

The procedure progressively progressed toward a more prominent and shorter incision while maintaining the detrusor muscle's posterior wall and honoring the inert mucosa's valve mechanism, which relaxes in response to the filling bladder's pressure [6]. This is the idea behind Rodriguez *et al*. [6] “smiling mouth” endoscopic meatotomy technique. Rodriguez claims that endoscopic meatotomy was also successfully performed with a Nd-YAG type laser by Gupta *et al*. [7] and a Ho-YAG type by Mazo *et al*. [8]. Liu *et al*. [9] employed the endoscopic technique outlined by Rodriguez in our observation, followed by the Ho-YAG laser's fragmentation of the stone.

## Conclusion

A rare congenital malformation called ureterocele is typically diagnosed in children and is rarely discovered in adults, necessitating sometimes lengthy diagnostic testing. Adult ureterocele treatment is not standardized. But for the time being, endoscopic meatotomy is still the recommended course of action.
